# Angiographic embolization followed by piecemeal resection of giant posterior mediastinal schwannoma: Case report and concise review

**DOI:** 10.1016/j.ijscr.2018.10.055

**Published:** 2018-10-29

**Authors:** Tyler J. Loftus, Mauricio Pipkin, Tiago Machuca, Olusola Oduntan

**Affiliations:** Department of Surgery, University of Florida, Gainesville, FL, United States

**Keywords:** Case report, Schwannoma, Angiographic embolization, Resection, Thoracic surgery, Thoracotomy

## Abstract

Introduction: Posterior mediastinal masses present unique diagnostic and therapeutic challenges, particularly when large highly vascularized tumors extend toward or emanate from the spinal cord. The rare nature of these tumors precludes the development of standardized management algorithms, underscoring the importance of case reports.

Presentation of case: A 57 year old female presented with exertional dyspnea and right chest pressure. Chest radiography followed by computed tomography (CT) scan demonstrated a 13 cm posterior mediastinal mass involving the T7 vertebral body. CT-guided percutaneous biopsy confirmed benign schwannoma. During open exploration, the tumor bled easily with contact. Angiography with intercostal arterial embolization decreased tumor vascularity while preserving spinal cord perfusion. Subsequent piecemeal resection facilitated exposure of the tumor base and complete resection. Postoperative recovery was uneventful.

Discussion: Neurogenic tumors are most commonly located in the posterior mediastinum. When untreated, schwannomas continue to grow, and will inevitably cause compressive symptoms if given sufficient time. Therefore, resection is recommended. This may be performed thoracoscopically in select patients with small tumors, avoiding the morbidity of a thoracotomy incision.

Conclusion: Large posterior mediastinal schwannomas require posterolateral thoracotomy and resection. Preoperative angiography helps identify arteries shared by the tumor and the spinal cord, and embolization may reduce tumor vascularity and operative blood loss thereby permitting safer resection.

## Highlights

• Radiographic assessment of spinal cord involvement is essential.
• Histology is necessary to establish etiology, prognosis, and treatment plan.
• Large tumors may require posterolateral thoracotomy and piecemeal resection.
• Preoperative angiography may identify arteries shared by the tumor and spinal cord.
• Preoperative angioembolization may reduce tumor vascularity and operative blood loss.

## 1 Introduction

Neurogenic tumors comprise approximately 80% of all posterior mediastinal tumors, typically arising from the spinal cord, sympathetic ganglia, or peripheral nerve roots [1]. Posterior mediastinal schwannomas originate from neural crest cells and typically arise from intercostal nerves [2]. Given their slow rate of growth and benign behavior, they are often discovered incidentally on imaging studies. Rarely, posterior mediastinal schwannomas present with compressive symptoms among patients who have not had recent thoracic imaging for any indication. The rare nature of these tumors precludes the development of standardized management algorithms, underscoring the importance of case reports as educational tools.

We present a case of a symptomatic posterior mediastinal schwannoma in which the late presentation and large tumor size necessitated intercostal artery embolization followed by thoracotomy with piecemeal resection. This work was performed in accordance with SCARE criteria [3]. Written informed consent was obtained from the patient. This study was exempt from approval by the University of Florida Institutional Review Board.

## 2 Presentation of case

**Fig. 1:**
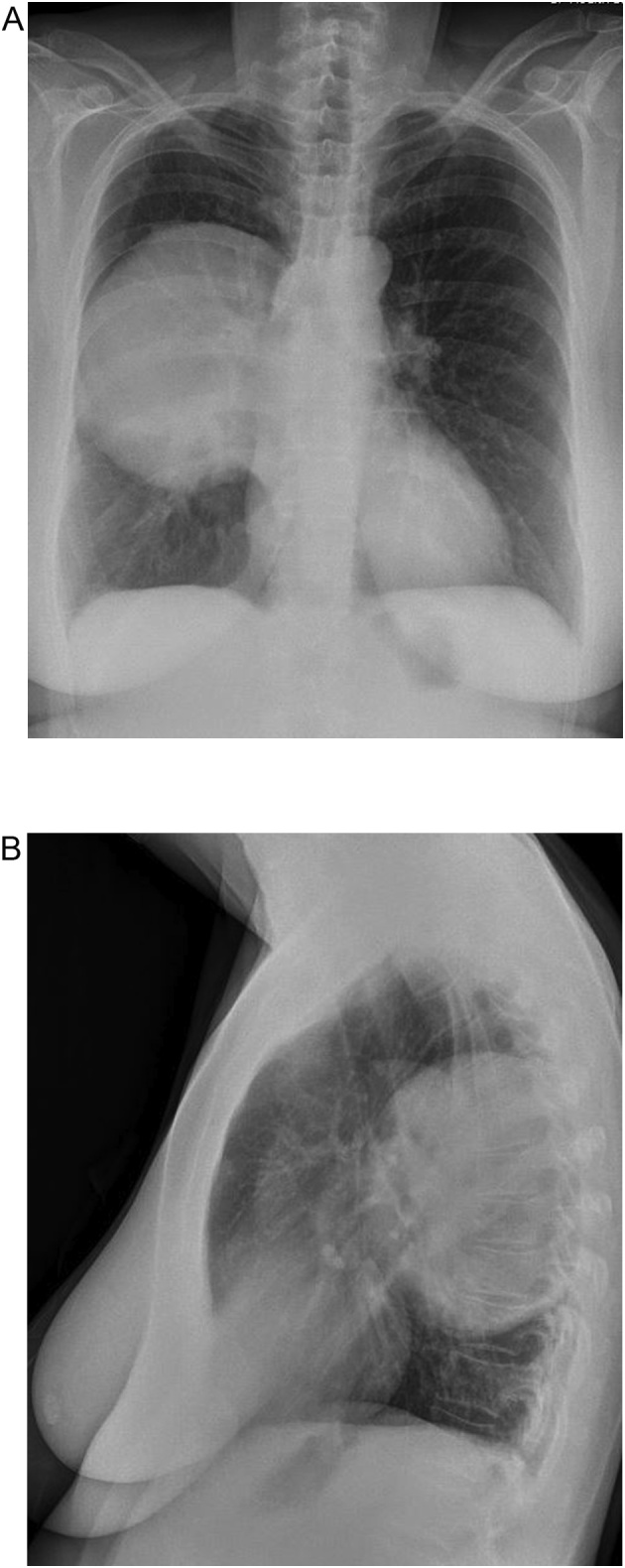
Chest radiograph demonstrating a large round opacity in the right chest (1A: Anterior-posterior view, 1B: Lateral view).

**Fig. 2:**
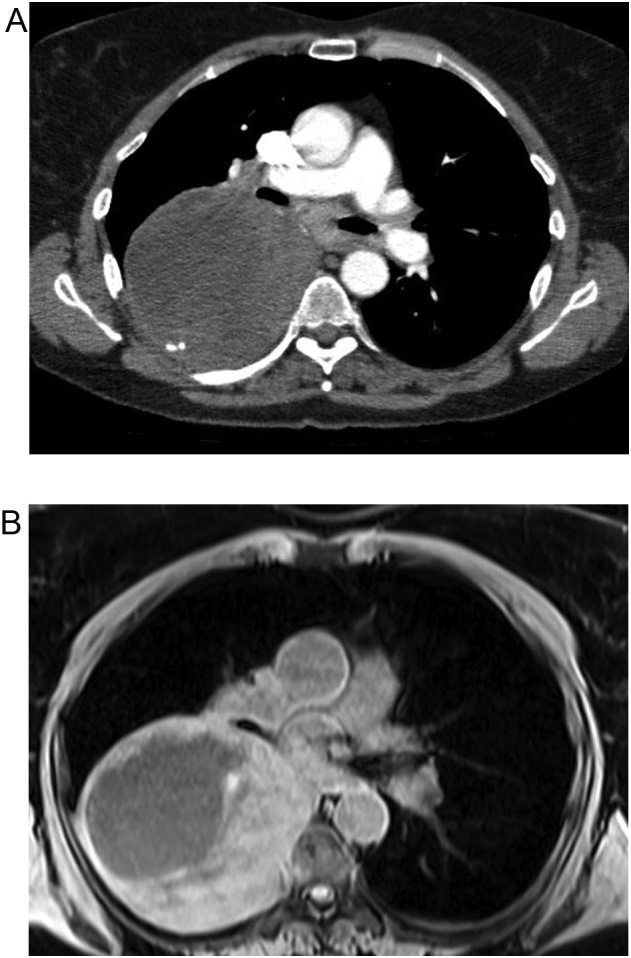
A: Computed tomography of the chest demonstrating a 13 cm pleural-based mass with focal areas of necrosis and arterial enhancement. B: Magnetic resonance imaging of the chest demonstrating focal extension of the mass through the parietal pleura into the paravertebral space and into the T7 vertebral body without encroachment into the spinal canal or evidence of spinal cord compression.

The patient is a 57 year old female with no underlying medical conditions or daily medications who had smoked one pack of cigarettes daily for ten years and had no family history of benign or malignant thoracic tumors. She initially presented to an emergency department with exertional dyspnea and right chest pressure exacerbated by dry cough. Chest radiography demonstrated a large round opacity in the right chest (Fig. 1), prompting computed tomographic (CT) imaging of the chest, which demonstrated a 13 cm pleural-based mass in the posterior mediastinum with focal areas of central necrosis and arterial enhancement (Fig. 2A). The mass appeared to involve the T7 vertebral body. Magnetic resonance imaging of the chest demonstrated focal extension through the parietal pleura into the paravertebral space and into the T7 vertebral body without encroachment into the spinal canal or evidence of spinal cord compression (Fig. 2B). There was a well-corticated margin at the site where the mass was invading the vertebral body, suggesting a chronic pressure-type remodeling process. Her neurologic examination was normal.

CT-guided core needle biopsy of the mass demonstrated a spindle cell neoplasm consistent with schwannoma. She was subsequently referred to us for Thoracic Surgery evaluation. Elective operative resection was recommended, primarily for symptom relief.

**Fig. 3:**
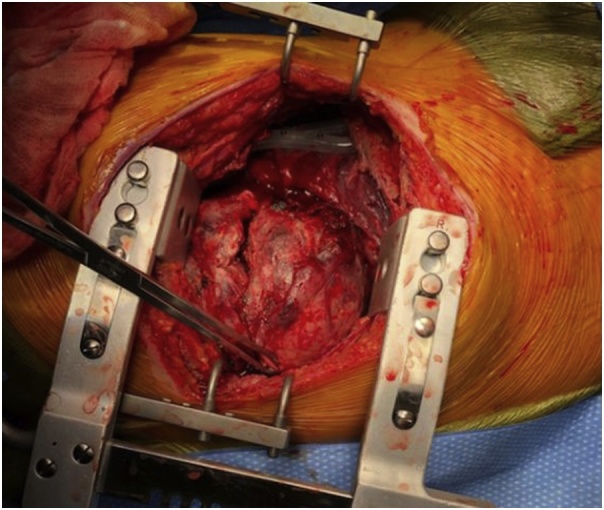
Intra-operative finding: large tumor occupying right hemithorax.

A right posterolateral thoracotomy was performed at the sixth intercostal space, and a short segment of the seventh rib was resected. A large posterior mediastinal mass occupying most of the lower portion of the chest and causing compressive atelectasis of the entire right lower lobe was encountered (Fig. 3). During attempts at separating the mass from the right lower lobe, the mass bled easily with contact. To facilitate tumor handling and mobilization, fluid was aspirated from the center of the mass. However, continued diffuse contact hemorrhage precluded safe dissection. Therefore, silk sutures were used to ligate visible vessels on the surface of the capsule of the mass and the chest was closed with plans for angiographic embolization of the intercostal vessels perfusing the mass.

**Fig. 4:**
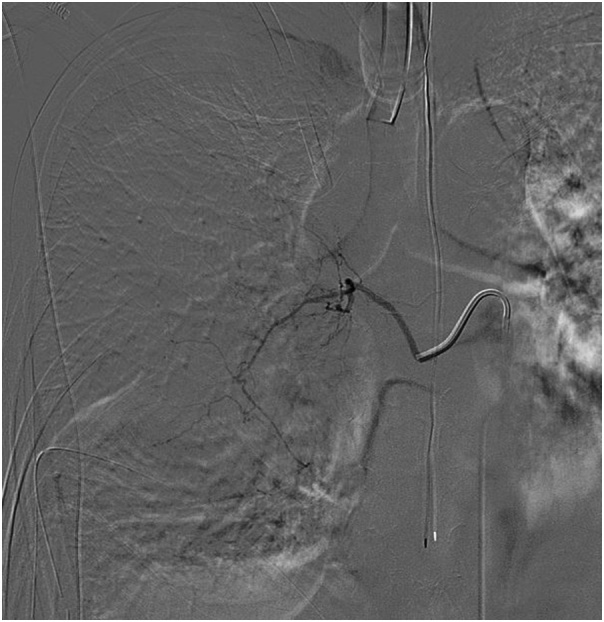
Angioembolization of intercostal arteries perfusing the mass.

The following day, the patient was taken to the interventional radiology suite for descending thoracic aortogram. It was noted that the mass was perfused by right T7-T10 intercostal arteries. Particle embolization of these arteries was performed (Fig. 4). The artery of Adamkiewicz was visualized at T11 and it was preserved to maintain spinal cord perfusion.

Four days following angio-embolization, the patient returned to the operating room for re-exploration. The seventh rib was resected to facilitate exposure. In contrast to the diffuse contact hemorrhage observed with gentle manipulation of the mass during the first operation, the mass now remained relatively hemostatic during dissection. To allow excision without placing excessive traction on the stalk and risk for avulsion injury to the vascular pedicle, the mass was excised completely in a piecemeal fashion. This operation, as well as the initial operation, was performed by board-certified thoracic surgeons at a university teaching hospital.

**Fig. 5:**
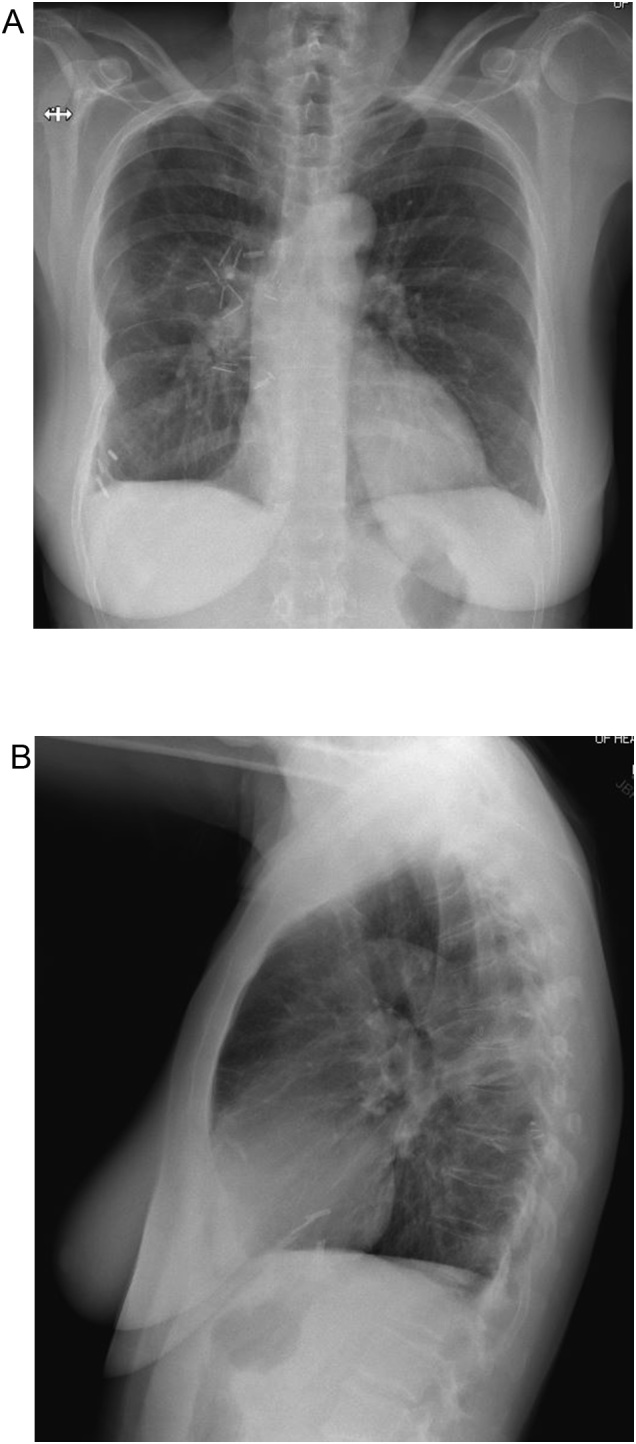
Post-operative chest radiograph (1A: Anterior-posterior view, 1B: Lateral view).

Pain control was achieved with use of thoracic epidural catheter. Her post-operative course was uneventful. Chest tubes were removed on third day and she was discharged home a day later in good condition. She returned to clinic one month following discharge. Apart from on-going requirement for non-steroidal anti-inflammatory and opiate medications for pain control, she was recovering well. She verbalized her gratitude for the care provided and the relief of her dyspnea. Her chest radiograph at follow up showed clear lung fields with complete resolution of pulmonary edema (Fig. 5).

## 3 Discussion

Schwannomas, like other neurogenic tumors, typically arise in the posterior mediastinum. However, they may also arise from the middle mediastinum, and have been reported to arise from the phrenic nerve, recurrent laryngeal nerve, vagus nerve, and pulmonary artery [[4], [5], [6], [7]]. Because anatomic location provides only a general approximation, other radiographic characteristics are important elements of the diagnostic workup. On CT scan, schwannomas appear as well-circumscribed masses that are heterogeneous following intravenous contrast administration, with hypodense areas likely representing cystic degeneration [8]. On MRI, schwannomas appear heterogeneous with T2 hyperintensity representing cystic degeneration. Schwannoma fluorodeoxyglucose (FDG) uptake is variable, limiting the utility of FDG-positron emission tomography [9].

Because imaging characteristics of mediastinal masses often do not allow for definitive diagnosis and may not differentiate benign from malignant conditions, histopathologic assessment is prudent. Depending on the location of the tumor, this may be accomplished with fine-needle aspiration, ultrasound or CT-guided percutaneous biopsy, mediastinotomy, or video-assisted thoracoscopic surgery [10]. However, less invasive approaches are preferred to reduce associated morbidity [11].

On histologic assessment, schwannomas contain areas of dense spindle cells (Antoni A) and hypocellular stromal areas (Antoni B), and exhibit protein S100 positivity [12].

When untreated, schwannomas continue to grow, and will inevitably cause compressive symptoms if given sufficient time. Despite the low likelihood of malignant transformation, resection of mediastinal schwannomas is advised, unless the patient has a short life expectancy or severe comorbidities placing them at substantial risk for perioperative and postoperative complications. When feasible, thoracoscopic approaches minimize the pain and morbidity associated with a thoracotomy incision [[13], [14], [15]]. However, giant masses may not allow sufficient working space for safe thoracoscopic resection. In such cases, posterolateral thoracotomy is recommended, and rib resection may facilitate exposure and minimize the risk of unintentional and uncontrolled fractures secondary to excessive force placed by self-retained retractors. Even with this approach, piecemeal resection of the mass may be necessary to allow exposure of the tumor base.

Angioembolization has the potential to decrease the vascularity of the tumor and decrease intraoperative blood loss. Madariaga et al. [16] recently reported a 14-year experience from the Massachusetts General Hospital in which preoperative angiography was employed for ten patients with posterior mediastinal tumors. This approach allowed for recognition of aberrant arterial anatomy and associated spinal arteries as well as embolization in seven of the ten patients. Compared with patients with posterior mediastinal masses who did not undergo preoperative angiography during the same time period, patients who had angiography had larger tumors, were more likely to have neuroforamen involvement, and had longer hospital length of stay, which may have been attributable to the anatomical characteristics of the tumors managed with preoperative angiography. Because angiography prior to embolization facilitates identification of spinal arteries, spinal cord ischemia is avoidable, but may preclude embolization for patients with blood supply shared by the tumor and spinal cord. Unfortunately, limited sample sizes and individual institution experience hinders the development of standardized protocols to select patients with posterior mediastinal masses for preoperative angiography. However, it seems prudent to consider preoperative angiography and embolization as indicated for patients with large tumors at increased risk for large volume intraoperative hemorrhage, particularly when neuroforamen involvement increases the complexity of the dissection.

## 4 Conclusion

Angiography prior to resection of large schwannomas is useful for identifying tumor blood supply, aberrant vascular anatomy, and arteries shared by the tumor and spinal cord. Intercostal artery embolization serves to reduce tumor vascularity and operative blood loss. Pursuing this approach would most likely have spared our patient the intraoperative blood loss incurred at the first operation, and the need for a second surgery.

Future research should seek to establish standardized protocols to select patients with posterior mediastinal masses for preoperative angiography.

## Conflicts of interest

None.

## Funding

None.

## Ethical approval

This study was exempt from approval by the University of Florida Institutional Review.

## Consent

Written informed consent was obtained from the patient. This study was exempt from approval by the University of Florida Institutional Review.

## Author contribution

TJL and OO contributed to study conception and design. All authors contributed to the literature review. TJL drafted the manuscript. MP, TM, and OO provided critical revisions. All authors approved the final version of the manuscript.

## Registration of research studies

A case report is a medical/educational activity that does not meet the Department of Health and Human Services definition of “research”, which is: "a systematic investigation, including research development, testing and evaluation, designed to develop or contribute to generalizable knowledge."

## Guarantor

TJL and OO accept full responsibility for this work.

## Provenance and peer review

Not commissioned, externally peer reviewed.
